# Supplementary material for: Resolving the full spectrum of human genome variation using Linked-Reads
Source: Genome Res. 2019 Apr;29(4):635–45. doi: 10.1101/gr.234443.118 (PMC6442396; doi:10.1101/gr.234443.118)
Supplement: Supplemental Material [file supp_gr.234443.118_Supplemental_longranger-2.2.zip › longranger-2.2.tar.bz2!/lr2.2-public/lib/stan/cmdstan/stan/doc/api/html/structstan_1_1math_1_1promote__scalar__type_3_01_t_00_01_eigen_1_1_matrix_3_01_s_00_01_eigen_1_1e90c042d64d8c3a6b9383f570932df58.html]

Stan: stan::math::promote\_scalar\_type< T, Eigen::Matrix< S, Eigen::Dynamic, Eigen::Dynamic > > Struct Template Reference


|  |  |
| --- | --- |
|  | Stan  2.5.0  probability, sampling & optimization |


- Main Page
- Related Pages
- Namespaces
- Classes
- Files

- Class List
- Class Index
- Class Hierarchy
- Class Members

All Classes Namespaces Files Functions Variables Typedefs Enumerations Enumerator Friends Macros Pages

- stan
- math
- promote\_scalar\_type< T, Eigen::Matrix< S, Eigen::Dynamic, Eigen::Dynamic > >

Public Types |
List of all members

stan::math::promote\_scalar\_type< T, Eigen::Matrix< S, Eigen::Dynamic, Eigen::Dynamic > > Struct Template Reference

Template metaprogram to calculate a type for a matrix whose underlying scalar is converted from the second template parameter type to the first.
More...

`#include <promote_scalar_type.hpp>`

|  |  |
| --- | --- |
| Public Types | |
| typedef Eigen::Matrix  < typename promote\_scalar\_type  < T, S >::type, Eigen::Dynamic,   Eigen::Dynamic > | type |
|  | The promoted type. More... |
|  | |

## Detailed Description

### template<typename T, typename S> struct stan::math::promote\_scalar\_type< T, Eigen::Matrix< S, Eigen::Dynamic, Eigen::Dynamic > >

Template metaprogram to calculate a type for a matrix whose underlying scalar is converted from the second template parameter type to the first.

This is the case for a vector container type.

Template Parameters
:   |  |  |
    | --- | --- |
    | T | result scalar type. |
    | S | input matrix scalar type |

Definition at line 23 of file promote\_scalar\_type.hpp.

## Member Typedef Documentation

template<typename T , typename S >

typedef Eigen::Matrix<typename promote\_scalar\_type<T,S>::type, Eigen::Dynamic, Eigen::Dynamic> stan::math::promote\_scalar\_type< T, Eigen::Matrix< S, Eigen::Dynamic, Eigen::Dynamic > >::type

The promoted type.

Definition at line 30 of file promote\_scalar\_type.hpp.

---

The documentation for this struct was generated from the following file:

- src/stan/math/matrix/promote\_scalar\_type.hpp


---

[ Stan Home Page ]

*© 2011–2014,
Stan Development Team.*
